# Genomic Epidemiology and Evolution of Diverse Lineages of Clinical Campylobacter jejuni Cocirculating in New Hampshire, USA, 2017

**DOI:** 10.1128/JCM.02070-19

**Published:** 2020-05-26

**Authors:** Cooper J. Park, Jinfeng Li, Xinglu Zhang, Fengxiang Gao, Christopher S. Benton, Cheryl P. Andam

**Affiliations:** a University of New Hampshire, Department of Molecular, Cellular and Biomedical Sciences, Durham, New Hampshire, USA; b New Hampshire Department of Health and Human Services, Concord, New Hampshire, USA; National Institute of Allergy and Infectious Diseases

**Keywords:** *Campylobacter jejuni*, genomic epidemiology, antibiotic resistance, virulence, recombination

## Abstract

Campylobacter jejuni is one of the leading causes of bacterial gastroenteritis worldwide. In the United States, New Hampshire was one of the 18 states that reported cases in the 2016 to 2018 multistate outbreak of multidrug-resistant C. jejuni. Here, we aimed to elucidate the baseline diversity of the wider New Hampshire C. jejuni population during the outbreak. We used genome sequences of 52 clinical isolates sampled in New Hampshire in 2017, including 1 of the 2 isolates from the outbreak. Results revealed a remarkably diverse population composed of at least 28 sequence types, which are mostly represented by 1 or a few strains. A comparison of our isolates with 249 clinical C. jejuni from other states showed frequent phylogenetic intermingling, suggesting a lack of geographical structure and minimal local diversification within the state. Multiple independent acquisitions of resistance genes from 5 classes of antibiotics characterize the population, with 47/52 (90.4%) of the genomes carrying at least 1 horizontally acquired resistance gene. Frequently recombining genes include those associated with heptose biosynthesis, colonization, and stress resistance. We conclude that the diversity of clinical C. jejuni in New Hampshire in 2017 was driven mainly by the coexistence of phylogenetically diverse antibiotic-resistant lineages, widespread geographical mixing, and frequent recombination. This study provides an important baseline census of the standing pangenomic variation and drug resistance to aid the development of a statewide database for epidemiological studies and clinical decision making. Continued genomic surveillance will be necessary to accurately assess how the population of C. jejuni changes over the long term.

## Abstract (precis)

Campylobacter jejuni is one of the leading causes of bacterial gastroenteritis worldwide. In the United States, New Hampshire was one of the 18 states that reported cases in the 2016 to 2018 multistate outbreak of multidrug-resistant C. jejuni. Here, we aimed to elucidate the baseline diversity of the wider New Hampshire C. jejuni population during the outbreak. We used genome sequences of 52 clinical isolates sampled in New Hampshire in 2017, including 1 of the 2 isolates from the outbreak.

## INTRODUCTION

Campylobacter jejuni is a major foodborne pathogen and the most commonly reported bacterial cause of gastroenteritis (campylobacteriosis) in the United States and worldwide (1, 2). Severe cases of C. jejuni infections can also lead to invasive infections, such as bacteremia (3). Infection with C. jejuni is also considered one of the main precedents for the development of the autoimmune condition Guillain-Barré syndrome (GBS), a serious demyelinating neuropathy (4). The World Health Organization estimates that *Campylobacter* spp. have resulted in 166 million illnesses and 37,604 deaths in 2010 worldwide (1). In the United States, the Centers for Disease Control and Prevention (CDC) estimates a total of 1.5 million infections and $270 million in direct medical costs every year caused by *Campylobacter* infections, mostly involving C. jejuni (5). Because *Campylobacter* sp. naturally colonizes the gastrointestinal tract of food-producing, companion, and wild animals, disease outbreaks have often been linked to the consumption of raw, undercooked, or contaminated water, food, and food products as well as through direct contact with animals (6).

Due to the self-limiting characteristics of campylobacteriosis, antimicrobial therapy is not routinely recommended; however, in acute or persistent infections, immunocompromised cases, or those patients with comorbidities, antibiotics are commonly prescribed (6). The emergence and spread of *Campylobacter* isolates exhibiting resistance to antibiotics commonly used to treat severe infections have been alarmingly increasing in the past two decades (5, 7, 8). In CDC’s 2019 report on Antibiotic Resistance Threats in the United States, antibiotic-resistant *Campylobacter* sp. is listed as 1 of the 11 serious threats to public health that require prompt and sustained action (5). The CDC estimates that 28% of *Campylobacter* isolates from 2015 to 2017 have decreased susceptibility to ciprofloxacin (fluoroquinolone), 4% with decreased susceptibility to azithromycin (macrolide), and 2% with decreased susceptibility to both ciprofloxacin and azithromycin (5). The public health threat of antibiotic resistance (ABR) in this pathogen was recently brought to light when a multistate outbreak of multidrug-resistant C. jejuni infections occurred in the United States from January 2016 to February 2018 (9). The source of the outbreak were puppies from breeders, distributors, and pet stores (9). Antibiotic susceptibility testing showed that the outbreak isolates were resistant to all antibiotics commonly used to treat *Campylobacter* infections (9). The state of New Hampshire was 1 of the 18 states that reported cases in the 2016 to 2018 C. jejuni outbreak, with 2 of the 118 cases reported (9). In our study, we aimed to elucidate the genetic diversity of the wider New Hampshire C. jejuni population during the period of the outbreak, how resistance and virulence determinants are distributed among strains, and the evolutionary processes that have shaped the local population. This study provides an important baseline census of the standing C. jejuni pangenomic diversity and drug resistance characteristics in New Hampshire to aid in the development of a statewide database for epidemiological studies and clinical decision making. Continued genomic surveillance of the background diversity will be necessary to accurately assess how the population of C. jejuni changes over the long term, in response to changes in the selective landscape, and during disease outbreaks.

## MATERIALS AND METHODS

### Bacterial isolates.

Isolates were submitted to the Public Health Laboratories, New Hampshire Department of Health and Human Services (NH DHHS) in Concord, NH, in 2017. These isolates were received from New Hampshire health care providers and were recovered primarily from stool specimens collected from individuals with *Campylobacter* infection. The state of New Hampshire considers *Campylobacter* infections as a reportable disease and the NH DHHS strongly encourages isolate submission to the Public Health Laboratories. However, submission of isolates is not mandatory. No identifiable information is associated with the isolates submitted by the health care providers. In total, our data set comprised 52 isolates.

### DNA extraction and genome sequencing.

Sequencing of *Campylobacter* isolates is part of the PulseNet surveillance program, a United States national laboratory network that connects foodborne illness cases to detect outbreaks (10). DNA extraction, library preparation, and whole-genome sequencing were done following the PulseNet standard operating procedures (https://www.cdc.gov/pulsenet/pathogens/wgs.html). Briefly, DNA extraction procedures were conducted using the DNeasy blood and tissue kit (Qiagen, Valencia CA). DNA quality and concentration were measured using Qubit fluorometer and NanoDrop spectrometer. A total of 1 ng of genomic DNA from each isolate was used to construct sequencing libraries using the Illumina Nextera XT DNA library preparation kit (Illumina, Inc. San Diego, CA) per the manufacturer’s instructions. Samples were sequenced as multiplexed libraries on the Illumina MiSeq platform operated per the manufacturer’s instructions for 500 cycles to produce paired-end reads of 250 bp in length. The MiSeq sequencer is housed at the NH DHHS Public Health Laboratories.

### *De novo* genome assembly, annotation, pangenome, and phylogenetic analyses.

We used the Nullarbor pipeline v2.0 (https://github.com/tseemann/nullarbor) to perform read trimming, quality assessment, contig assembly, gene annotation, pangenome analysis, sequence type (ST) identification, sequence alignment, and phylogenetic analysis of the entire data set. The Nullarbor pipeline can be described as follows: adapters were trimmed using Trimmomatic v0.38 (11). Trimmed reads were assembled into contigs using SKESA v2.3.0 (12) using a C. jejuni subsp. *jejuni* reference genome obtained from the NCBI RefSeq database (accession no. GCF_000009085.1). The quality of genome assemblies was assessed using QUAST (13). Assembled genomes were annotated using Prokka v1.13.3 (14) with default parameters. Roary v3.12.0 (15) was used to characterize the pangenome of the New Hampshire C. jejuni data set and to classify genes into core, soft core, shell, and cloud genes. Each orthologous gene family was aligned using MAFFT v7.407 (16). The ST of each isolate was determined using the program multilocus sequence typing (MLST) (https://github.com/tseemann/mlst), which extracts the sequences of seven housekeeping genes (*aspA*, *glnA*, *gltA*, *glyA*, *pgm*, *tkt*, and *uncA*) from the Illumina raw data and compares them to the C. jejuni MLST database (17). Single nucleotide polymorphisms (SNPs) from the core genes were identified and aligned using Snippy v4.3.6 (https://github.com/tseemann/snippy) and were used to generate a maximum likelihood phylogeny using the program IQ-TREE v1.6.9 (18).

To determine the degree of overall genomic relatedness between genomes, we calculated the genome-wide average nucleotide identity (ANI) for all possible pairs of genomes using the program FastANI v1.0 (19). ANI estimates the average nucleotide identity of all orthologous genes shared between any two genomes (19). Organisms belonging to the same species typically exhibit ≥95% ANI (19). Pairwise ANI values were visualized using an heatmap generated in R (20) and the ggplot2 package (21).

To place the New Hampshire isolates within a country-wide context, we queried the genome sequences of 48,987 clinical C. jejuni isolates that were included in the 100K Pathogen Project as of March 2020 (22). Of these isolates, we selected 367 isolates that were derived only from the United States, from human samples, from clinical specimens, as well as those that have information on their state of origin. These isolates were filtered further to include only those genomes that are within the 95% ANI threshold that defines a bacterial species (19). A total of 249 genomes representing 13 other states were used for comparison with the New Hampshire genomes (see Table S1 in the supplemental material). After annotating with Prokka (14) and identifying the pangenome using Roary (15), we generated a core genome phylogeny using RAxML v8.2.11 (23) with a general time-reversible nucleotide substitution model, 4 gamma categories for rate heterogeneity, and 100 bootstrap replicates.

### *In silico* identification of ABR genes, virulence genes, and plasmids.

We screened all genomes for known resistance and virulence genes using a direct read mapping method called ABRicate v0.8.10 (https://github.com/tseemann/abricate) implemented in Nullarbor. ABRicate identifies ABR genes using a BLASTN comparison search (24) against the Resfinder database (25). ABRicate identifies only horizontally acquired resistance genes and not resistance due to chromosomal mutations. Virulence genes were identified using BLASTN against the Virulence Factor Database (VFDB) (26). Some of these predicted genes may be complete, exact matches, or incomplete; hence, ABRicate classifies the predicted genes based on the proportion of the gene that is covered. These categories are present (≥95% sequence coverage), questionable (<95% sequence coverage), and absent, which provide a level of confidence on ABRicate’s predictions. We also used PlasmidFinder with default parameters to perform an *in silico* detection and characterization of plasmid sequences (27).

### Recombination detection.

Using the core genome alignment, we calculated the pairwise homoplasy index test implemented in SplitsTree v4.14.8 (28) to determine the statistical likelihood of recombination being present in the entire data set (29). This statistic measures the genealogical correlation or similarity of adjacent nucleotide sites. Under the null hypothesis of no recombination, the genealogical correlation of adjacent sites is invariant to permutations of the sites because all sites should have the same evolutionary history (29). The significance of the observed index was estimated using a permutation test. We then visualized potential recombination events using SplitsTree, which integrates reticulations due to recombination in a phylogeny (28). To identify the most frequently recombining genes across the genomes, we used fastGEAR (30) with default parameters on individual core and shared accessory genes identified by Roary. To test the significance of the inferred recombination events and identify false positives, we used the diversity test implemented in fastGEAR, which compares the diversity of the recombined fragment in question to its sequence background. Recombinations were visualized using R (20) and the postprocessing scripts provided by fastGEAR. We used EggNOG-mapper v2 to perform orthology assignment for functional annotation of the recombined genes (31). The reference sequences of recombined genes were used as inputs to obtain the Gene Ontology identities (IDs). We restricted our search to only within the subphylum *Epsilonproteobacteria* to which the genus *Campylobacter* belongs. These IDs were then used as input in the Web tool PANTHER (32) to perform a statistical overrepresentation test to determine if the recombined genes were biased toward a specific ontological process. PANTHER classifies the ontological function of each recombined gene using different categories, namely molecular function, cellular component, biological process, and protein class.

Parameters used for all programs are listed in Table S1.

### Data availability.

All *Campylobacter* genomic sequences generated under PulseNet surveillance (10) are uploaded in real-time to the Sequence Read Archive (SRA) hosted by the National Center for Biotechnology Information (NCBI). The genome sequences analyzed in this study are available under BioProject accession number PRJNA239251. The genome sequences obtained from the 100K Pathogen Project were obtained from BioProject accession number PRJNA186441. Accession numbers and BioSample identifiers (IDs) are listed in Table S2 in the supplemental material.

## RESULTS

### Genomic characteristics of C. jejuni in New Hampshire.

**FIG 1:**
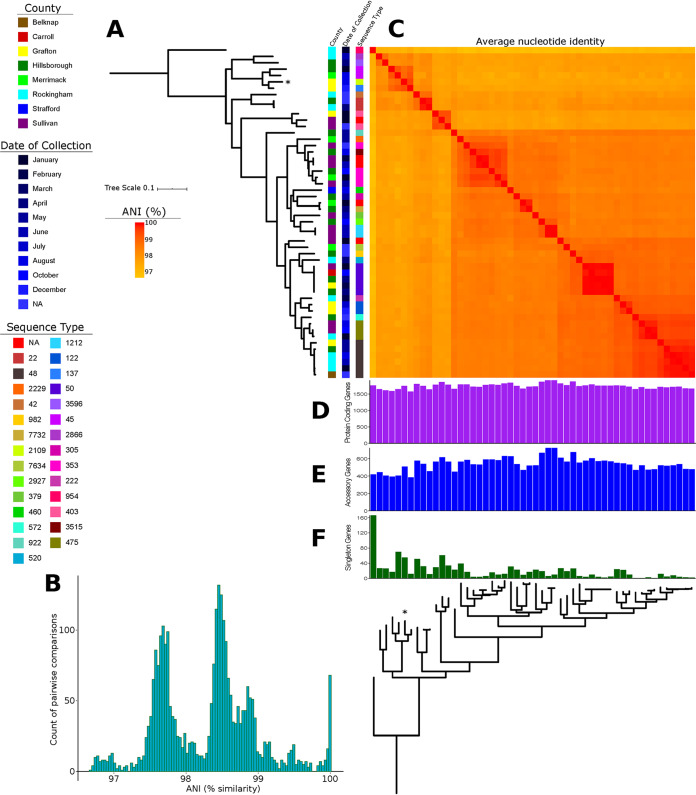
Phylogenetic relationships and pangenome characteristics of the 52 C. jejuni isolates. (A) The phylogeny was reconstructed using 83,210 core SNPs. The scale bar represents the number of nucleotide substitutions per site. The asterisk indicates the genome of the C. jejuni from the multistate puppy outbreak. (B) Frequency distribution of all pairwise ANI values. (C) ANI values were calculated for every pair of genomes in the entire data set. Bar plots show the number of protein coding genes (D), accessory genes (E), and singleton genes (F) per genome. Singleton genes are those that are unique to an individual genome.

We sequenced the genomes of 52 clinical C. jejuni isolates collected in New Hampshire in 2017 (Table S2). The genome sequences contain between 21 and 78 contigs, and *N*_50_ values range between 34,459 and 197,591 bp. *De novo* genome assemblies generated sequences of sizes ranging from 1.57 to 1.81 Mb (mean, 1.70 Mb). We used PlasmidFinder to determine if the variation in genome size could be attributed to the presence or absence of plasmids. No plasmids were detected in any of the New Hampshire genomes. We next used Roary to estimate the pangenome of the entire C. jejuni data set (see Fig. S1 and Table S3 in the supplemental material). Of the 4,335 gene families identified in the pangenome, a total of 1,176 genes comprised the core genome (genes present in 99% ≤ strains ≤ 100%), which represents approximately 27% of the pangenome. The maximum likelihood phylogenetic tree based on the alignment of 83,210 core SNPs revealed lineages that have relatively little structure relative to the location of the health care provider (county) or date of collection (Fig. 1A). Genome-wide ANI values for every possible pair of C. jejuni genomes ranged from 96.7% to 99.99% (mean, 98.26%) (Fig. 1B and C; see Table S4 in the supplemental material). The core genes (*n* = 1,176 genes) and the soft-core genes (*n* = 111 genes; genes present in 95% ≤ strains < 99%) constituted only 29.69% of the entire population’s pangenome. Accessory genes can be categorized into shell (*n* = 881; genes present in 15% ≤ strains < 95%) and cloud genes (*n* = 2,167; genes present in <15% of strains). Together, both categories of accessory genes constituted 70.31% of the population’s pangenome. There was substantial strain-level variation in the New Hampshire population in terms of gene content. The number of protein-coding genes per genome ranged from 1,575 to 1,918 (mean, 1,743) (Fig. 1D). The number of accessory genes per genome ranged from 385 to 724 (mean, 539.8) (Fig. 1E). Many accessory genes were unique to individual strains (1,059 genes, representing 24.42% of the pangenome), with 1 to 166 singleton genes present per genome (Fig. 1F).

Our results from *in silico* MLST showed that the C. jejuni isolates belonged to 28 unique known STs (Fig. 1A; see Table S5 in the supplemental material). Four novel STs found in five strains have MLST profiles that did not match known STs in the MLST database (17). We did not identify any one genome that dominated the entire population; instead, the population was composed of multiple STs represented only by one or a few strains. The most common STs were ST48 and ST50, which were represented by six and five strains, respectively. In this data set, we also included a genome (SRA accession number SRR6152533) from one of the two isolates from New Hampshire that was part of the 2016 to 2018 multistate puppy-associated outbreak of multidrug-resistant C. jejuni (9). This isolate has been identified as ST2109.

### Relationship of the New Hampshire C. jejuni isolates to the wider United States population.

**FIG 2:**
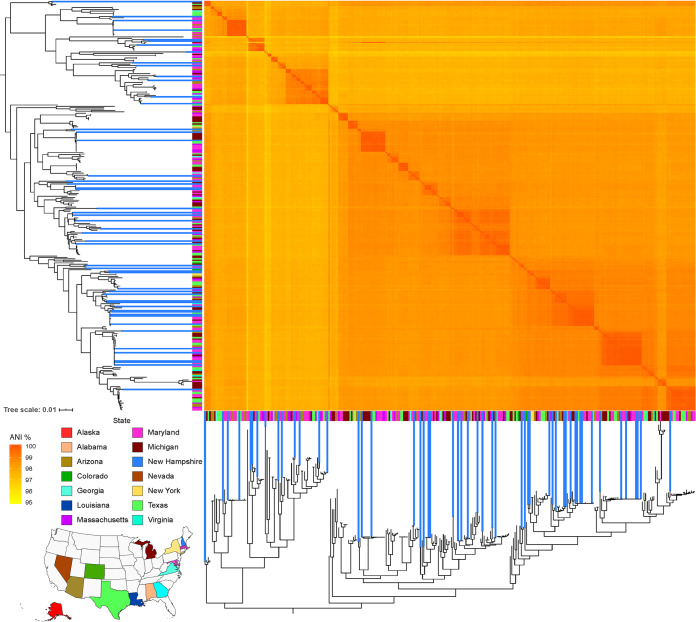
Phylogenetic relationships of our 52 C. jejuni isolates combined with 249 isolates from 13 other states in the United States. The genome sequences of the latter were obtained from the 100K Pathogen Project. The phylogeny was constructed from the alignment of 937 core genes. The scale bar represents the number of nucleotide substitutions per site. ANI values were calculated for every pair of genomes in the entire data set. The colored strip represents the state of origin for each isolate. The colored strips representing New Hampshire are elongated to distinguish them from the rest of the United States population.

To place the genetic diversity and population structure of the New Hampshire C. jejuni isolates within the broader United States C. jejuni population, we used a genome data set consisting of 249 clinical C. jejuni isolates primarily from stool specimens from the 100K Pathogen Project (Table S2) (22). These genomes represented 13 other states in the country. A pairwise genomic comparison in this merged data set (i.e., 52 isolates from New Hampshire and 249 from the 100K Pathogen Project) revealed ANI values that ranged between 96.06% and 100% (mean, 98.23%) (Fig. 2; Table S4). Pangenome analysis using Roary showed a total of 10,763 genes in the pangenome in the merged data set, which was 2.48 times more than the New Hampshire pangenome alone. We identified only 937 core and 203 soft-core genes, which were 0.2 times fewer and 1.8 times more than the New Hampshire pangenome, respectively. We also identified a total of 423 genes (representing 3.93% of the pangenome of the merged data set) that were found exclusively in the New Hampshire population compared with the 6,150 (representing 57.1% of the pangenome) found exclusively outside the state. A maximum likelihood tree generated using the alignment of the core genes showed that the phylogenetic clustering of isolates was independent of the state of origin and that the New Hampshire genomes were intermingled with those from other states (Fig. 2).

### Distribution of horizontally acquired ABR genes.

**FIG 3:**
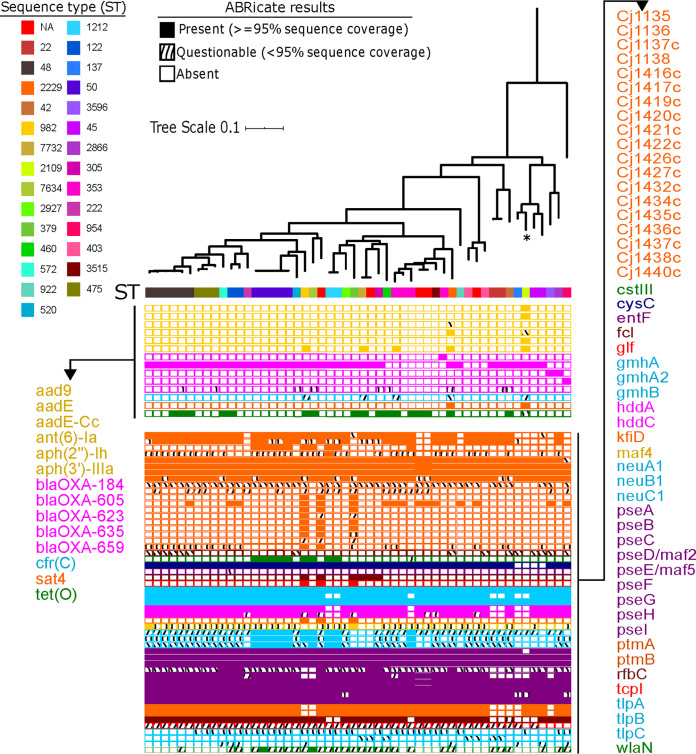
Summary of ABR and virulence profiles of individual C. jejuni genomes. Names of horizontally acquired resistance genes are on the left and are colored by antibiotic class. Names of virulence genes are listed on the right. Solid blocks indicate presence of gene (≥95% sequence coverage), wavy blocks indicate questionable presence (<95% sequence coverage), and empty boxes indicate the absence of the gene. The tree is identical to that in Fig. 1. Only those virulence genes that are differentially distributed among strains are shown here. A comprehensive list of all virulence genes identified in each strain is shown in Table S6.

Frequent horizontal gene transfer (HGT) characterizes the evolutionary history of numerous bacterial species (33), including *Campylobacter* members (34). In many bacterial pathogens, HGT has greatly contributed to the emergence and spread of many “superbugs” that have acquired resistance to a broad spectrum of antibiotics (35). We used the program ABRicate to determine the presence of horizontally acquired genes known to encode resistance to a range of different classes of antibiotics. We identified a total of 14 unique genes associated with ABR and which represent 5 different major classes of antibiotics (aminoglycosides, β-lactams, chloramphenicol-florfenicol, streptothricin, and tetracycline) (Fig. 3; see Table S6 in the supplemental material). Multiple independent acquisitions of resistance genes from the 5 major classes of antibiotics characterized the New Hampshire C. jejuni population, with 47/52 (90.4%) of the genomes carrying at least 1 horizontally acquired resistance gene. Five genomes (representing 9.6% of the population) carried at least one of the six genes that encode resistance against aminoglycosides. Of the 5 genes that encode β-lactam resistance, one gene (*bla*_OXA-605_) was found in 38 genomes, representing 73% of the population. Four other genomes harbored three other unique genes that encode β-lactam resistance. Overall, we found that resistance to β-lactams is most common in the population, with a remarkable 80.77% of the population carrying at least one of the five β-lactam resistance genes detected. Two genomes carried the *sat4* gene, which confers streptothricin resistance, while 17 genomes harbor the *tetO* gene which confers tetracycline resistance. One genome (SRA accession number SRR5859317) contained at least one resistance gene for each of the four classes (aminoglycosides, β-lactams, streptothricin, and tetracycline), while three genomes carried genes that encode resistance against three major classes of antibiotics. Notably, the isolate from the puppy outbreak shared at least three distinct ABR genes with the rest of the local population [*aph(3′)-IIIa*, *bla*_OXA-605_, and *sat4*] in addition to three other ABR genes that were unique to it [*aad9*, *aadE*, and *aph(2″)-Ih*]. It has been postulated that antibiotic use in puppies may have led to the emergence and transmission of multidrug-resistant C. jejuni isolates during the 2016 to 2018 outbreak (9). We also identified the likely presence of the multidrug resistance phenotype mediated by the plasmid-borne gene that encodes Cfr rRNA methyltransferase, which confers resistance to phenicols, lincosamides, oxazolidinones, pleuromutilins, and streptogramin A antibiotics (36, 37), in five genomes. Lastly, we did not detect the presence of any one acquired resistance gene in five genomes (9.6% of the population). Overall, we found that many of the clinical C. jejuni isolates in the local population were carriers of a diverse suite of resistance genes that can be horizontally exchanged between strains. The outbreak isolate was not the only one that was multidrug resistant; at least six other isolates carry transferrable genes that encode resistance against multiple classes of antibiotics.

### Distribution of virulence determinants.

We also used ABRicate to determine the presence of virulence genes in C. jejuni (Fig. 3; Table S6). In all, we detected a total of 126 virulence-related genes. A total of 78 virulence genes were most common in the population and were found in at least 50 out of 52 genomes. The most common virulence genes in the New Hampshire C. jejuni population were those that encode traits related to capsule, lipooligosaccharide, flagella-mediated motility, bacterial adherence to intestinal mucosa, invasive capability, toxin production, and type four secretion system. Genes associated with adherence included those that function in capsule variation, binding to fibronectin, lipooligosaccharide, and major outer membrane protein (porin) (26). Some virulence genes were particularly noteworthy and will be discussed here.

The cytolethal distending toxin (cdt) is one of the well-characterized virulence factors of C. jejuni and is reported to be associated with local acute inflammation in enterocolitis (38), hyperinvasion (39), and colorectal tumorigenesis (40). The C. jejuni *cdt* operon, consisting of *cdtA*, *cdtB*, and *cdtC*, encodes a multisubunit holotoxin that has DNase activity and induces DNA double-strand breaks (41, 42). While the presence of a single *cdt* gene does not have any effect on the virulence of C. jejuni, it has been reported that the presence of all three *cdt* genes results in the release of a functional cytotoxin (41). It is, therefore, not surprising that all three genes were found in at least 90% of the New Hampshire population, which consists solely of human clinical isolates. The *cdt* genes were present at high frequencies, namely *cdtA* in 51/52 genomes, *cdtB* in 47/52 genomes, and *cdtC* in 52/52 genomes. However, 1/52 and 5/52 genomes also possess *ctdA* and *cdtB*, respectively, but have <95% sequence coverage that may be due to sequencing errors.

C. jejuni is the most frequent pathogen associated with the acute immune-mediated neuropathies GBS and Miller-Fisher syndrome, which can cause acute flaccid paralysis in humans (4, 43). It has been previously reported that ganglioside mimicry by the C. jejuni lipooligosaccharide is a critical factor in eliciting the two neuropathies (4). The gene *wlaN* encodes β-1,3 galactosyltransferase, which is involved in the biosynthesis of ganglioside-mimicking lipooligosaccharide in C. jejuni (44). We detected *wlaN* in two genomes in the New Hampshire population. Previous studies on the prevalence of *wlaN* in C. jejuni from other geographical regions report similar low frequencies (e.g., 13% to 17% in 624 C. jejuni isolates from humans and poultry in Poland [45]; 7.5% in 58 stool isolates in Bangladesh [46]; 10% in 111 human, animal, and environmental isolates in Brazil [47]). In contrast, another study reported that, of the 40 isolates of C. jejuni from human, bovine, and turkey sources, *wlaN* was more prevalent and was detected in 46.7 % of strains that exhibit no or weak colonization and invasion capacity and in 60 % of strains with strong colonization and invasion capacity (48). Sialylated lipooligosaccharide has been reported to have the potential to also produce ganglioside mimics and induce GBS (49). The gene *cstIII*, which encodes a lipooligosaccharide sialyltransferase, is reported to also be associated with neuropathy (49). In the New Hampshire C. jejuni population, a total of nine strains carried the *cstIII* gene. For comparison, previous studies report the presence of *cstIII* in 30.8% of 266 isolates of human, chicken, bovine, and turkey origin in Germany (50) and in 18.9% of 827 genomes analyzed by the Food and Drug Administration Pacific Northwest Laboratory (49).

Glycosylation of *Campylobacter* flagellins with pseudaminic acid and its derivative has been previously shown to be essential for flagellar assembly and motility, which are required for colonization of the mucus lining of the gastrointestinal tract (51, 52). The genes *pseA* to *pseI* are required for the biosynthesis and/or transfer of pseudaminic acid to the flagellin (51, 52). In the New Hampshire population, we found that these genes were differentially distributed among genomes, namely 52/52 genomes have *pseB*, *pseC*, *pseF*, *pseG*, and *pseI*; 51/52 genomes have *pseA*; 7/52 genomes have *pseD*; 42/52 genomes have *pseE*; and 48/52 genomes have *pseH*. Such variation in the distribution of individual genes of an operon among closely related strains is not uncommon and may be indicative of frequent *in situ* gene displacement through gene gain and loss, which does not often result in losing the integrity and function of the operon (53). The differential distribution of these genes may also contribute to the generation of variation in flagellin glycosylation among strains that can influence antigenic diversity in C. jejuni (51).

### Reticulated evolution due to frequent recombination in New Hampshire genomes.

**FIG 4:**
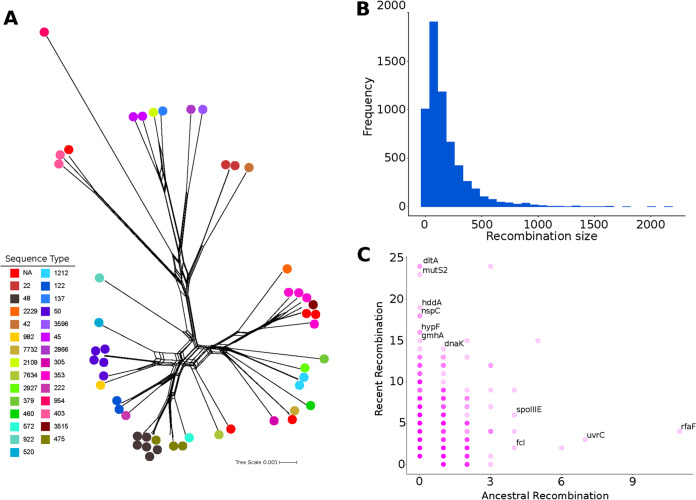
Recombination characteristics of the New Hampshire C. jejuni. (A) Phylogenetic SplitsTree network generated from the core genome alignment. The scale bar represents nucleotide substitutions per site. (B) Frequency distribution of the size of recombined DNA segments. (C) Genes that have undergone recent and/or ancestral recombination. For clarity, names of some of the most frequently recombined genes with known functions are shown. A list of all recombination events is presented in Table S7.

Recombination plays an important role in the evolutionary history of C. jejuni (54, 55). Here, we aimed to elucidate to what extent recombination contributes to the genomic structure of C. jejuni at the local scale. Using the pairwise homoplasy index statistic, we detected evidence for significant recombination in the core genome (*P* < 0.01). Recombination in the C. jejuni core genome can be visualized using NeighborNet implemented in SplitsTree4 (28), which showed the phylogenetic reticulations due to recombination (Fig. 4A). We then used fastGEAR to estimate recombination in core genes and shared accessory genes (30) (see Table S7 in the supplemental material). In the New Hampshire C. jejuni population, the lengths of the recombination fragments greatly varied. Overall, the sizes of recombination events followed a geometric distribution, with majority of the recombination encompassing short DNA segments and a median size of 116 bp (Fig. 4B). Large recombination events (>2,000 bp) occurred less frequently, with the longest recombination blocks detected in three genomes (SRA accession numbers SRR5278283 [ST475], SRR6014507 [ST48], and SRR6014981 [ST475]). Similar patterns of frequent microrecombinations and rare macrorecombinations (56) have been reported in other bacterial pathogens, such as Streptococcus pneumoniae and Salmonella enterica (56, 57). Such patterns have been reported to greatly contribute to shaping the genomic and phenotypic heterogeneity, including resistance and pathogenicity characteristics, of a pathogen species (56–58).

We also used fastGEAR to identify the genes that were frequently recombined. A total of 1,071 genes representing 24.7% of the pangenome have experienced recombination (Fig. 4C; Table S7). Of these genes, 1,020 were involved in recent recombination (i.e., recombination affecting a few strains) and 224 in ancestral recombination (i.e., recombination affecting entire lineages) (Fig. 4C). Some of the most frequently recombining genes with known function that fastGEAR detected included those that may contribute to virulence and adaptation. The gene product MutS2 has been reported to be associated with the overall function of preserving genomic integrity by inhibiting homologous recombination (59). The gene products of *hddA* (d-glycero-d-manno-heptose 7-phosphate kinase) and *gmhA* (phosphoheptose isomerase) are involved in heptose biosynthesis (60). Modifications in capsular heptose have been shown to contribute to C. jejuni colonization and persistence in the gastrointestinal tract (61). The gene product of *nspC* (carboxynorspermidine decarboxylase) is involved in the biosynthesis of the polyamine norspermidine, which functions in biofilm formation (62). The carbamoyltransferase encoded by *hypF* aids in the maturation of NiFe hydrogenases in Escherichia coli (63). *hypF* mutants have been shown to exhibit a loss of resistance against extreme acidic conditions (64), as in the case during passage through the stomach (65). Lastly, it is curious that *dltA* was identified as frequently recombining in the Gram-negative C. jejuni. The *dlt* operon functions in the d-alanylation of teichoic acids in Gram-positive bacteria and has been shown to confer resistance to antimicrobial peptides (66). A previous study reported the presence of the *dlt* operon in three Gram-negative genera (*Erwinia*, *Bordetella*, and *Photorhabdus*) and was thought to have been acquired by HGT (67).

To further elucidate the general functions of the recombined genes, we used EggNOG-mapper v2 and PANTHER to perform orthology prediction and functional annotation. Of the 1,071 genes inferred by Roary to have had experienced recombination, EggNOG-mapper v2 did not retrieve Gene Ontology results for 795 genes. Using PANTHER, we classified the remaining 276 genes based on different functional categories, namely molecular function, biological process, cellular component, and protein class (see Table S8 and Fig. S2 in the supplemental material). A total of 149 genes can be classified as having catalytic activity. A total of 131 genes were associated with metabolic processes. A total of 69 genes were associated with a variety of cellular components or the cytoplasm and 19 genes were associated with the cell membrane. Lastly, 137 genes were associated with metabolic interconversion enzymes. Overall, our recombination analysis shows that even within a single year of sampling, the standing pangenomic variation in a local population is amplified through frequent but variable recombination of genes associated with a variety of functions, which can greatly contribute to the potential of C. jejuni to evolve rapidly (34).

## DISCUSSION

Rapid advances and declining costs in whole-genome sequencing are transforming the public health system. Pathogen genomics is expected to become an integral part of a systematic surveillance required to monitor emerging trends in disease epidemiology, including campylobacteriosis, which will allow for earlier detection and more precise investigations of outbreaks, transmission, virulence, and drug resistance (68, 69). Pathogen genomic surveillance should include long-term monitoring of the standing pathogen diversity in any local population at a fine-scale resolution to provide a baseline census of antibiotic resistant and other high-risk clones circulating within a region, from local to global scales. Such information is integral in epidemiological studies and clinical decision making in managing *Campylobacter* infections. In this study, we analyzed the genomic diversity of 52 clinical isolates of C. jejuni in the state of New Hampshire in 2017. This data set was selected in order to assess the background genomic variation in C. jejuni during the 2016 to 2018 puppy-associated outbreak of multidrug-resistant C. jejuni in the United States. Our analysis included one of the two outbreak isolates that were reported in the state. Results revealed a remarkably high phylogenetic and genomic diversity of strains cocirculating in the wider New Hampshire C. jejuni population. Our results showed a lack of geographical structure and minimal local diversification within the state. We did not detect evidence for clonal expansion shaping the local population structure; the cocirculation of multiple STs suggests multiple introductions and widespread dissemination of divergent C. jejuni lineages between multiple counties in New Hampshire as well as between states, which may be facilitated by the constant movement of agricultural products, animals, and people.

The rapid evolution and diversification of C. jejuni within only a single year has also been facilitated by frequent recombination and HGT, which has been often observed in previous studies of C. jejuni (70–72). We present different lines of evidence to demonstrate the contribution of these processes in shaping the genomic structure of the New Hampshire population. First, we found that accessory genes are differentially distributed among strains, likely due to rapid gene gain and loss, which contribute to the overall genomic diversity of the local population. The variable distribution of accessory genes between strains is often attributed to adaptation to specific ecological niches (73, 74), even within the same host (75, 76). For example, mobile integrated elements and plasmids were reported to be more common in fecal than blood C. jejuni isolates, while a hybrid capsule locus was more common in blood than fecal isolates (77). Here, we show that even among fecal isolates, there is substantial heterogeneity in accessory gene content, which may indicate either neutral evolution due to random processes (78) or the existence of cryptic ecological niches (71) in the gastrointestinal tract that selects for certain adaptive genes. Second, the population harbors numerous horizontally acquired resistance determinants from five major classes of antibiotics. The origins and direction of transfer of these genes remain uncertain, but it is safe to assume that their acquisition and mobility may have greatly contributed to the overall distribution of ABR genes in the local population. The outbreak isolate has been previously characterized as multidrug resistant (9). Our analysis shows that it harbors six horizontally acquired resistance genes, three of which were unique to it and another three that were shared with other New Hampshire genomes. Yet, it is remarkable that the genome sequences of the rest of the population revealed that many of the isolates were also drug resistant, with resistance to beta-lactams being the most common. A few multidrug-resistant genomes were also detected. Hence, while the outbreak isolate did not spread through clonal expansion within the state, the risk of widespread dissemination of resistance genes through HGT among C. jejuni lineages is a serious public health threat and must be considered in the implementation of control measures and antibiotic stewardship practices. Lastly, frequently recombining genes include those associated with heptose biosynthesis, colonization, and stress resistance, of which all can have a substantial impact on the pathogen’s adaptive potential. This includes the rapid emergence of novel phenotypes (34, 79), such as multidrug resistance (80), and the ability to colonize a specific host (i.e., specialists) or multiple hosts (i.e., generalists) (55). Because increased genetic variation leads to more rapid adaptation (81), populations have a broader reservoir of mobile accessory genomic variants that can be mixed and matched in individual genomes through frequent recombination, which would suggest that individual strains each have a unique suite of capabilities to adapt to their environment.

Defining the baseline genomic diversity of a pathogen in a local population is integral to elucidating the ecological factors that sustain the cocirculation of diverse and drug-resistant lineages. It will aid in the development of a statewide database for epidemiological studies and clinical decisions in response to changing selective pressures and during disease outbreaks (68, 69). This is particularly important to precisely identify and trace high-risk clones in the local population that can disseminate easily or accumulate additional resistance mechanisms. While the 2017 genomes were phylogenetically diverse, represented by 28 unique known STs, it remains unclear whether there are certain lineages that will become more successful over the long term, e.g., hypervirulent, hyperrecombinant, highly transmissible, or multidrug resistant. Only continuous genomic surveillance of the local population over many years will allow us to determine the bacterial population dynamics within the state. Nevertheless, our study provides the initial genomic surveillance of C. jejuni for New Hampshire, which can be built on in future years to track the evolutionary changes that underlie phenotypic and population shifts of high-risk or superfit clones over time.

A few limitations need to be acknowledged. First, bacterial samples were based on what were received by the NH DHHS from local health providers and may not fully reflect the clinical C. jejuni diversity present in the entire state. It is likely that numerous and genetically distinct lineages in the clinical setting circulate in New Hampshire but remain undiscovered or undetected (e.g., if a strain causes less severe symptoms in a patient during infection and, thus, they may not seek medical intervention). The broad phylogenetic and pangenomic diversity of the New Hampshire population paired with a low sample size in this study suggests that we have merely touched on the existing diversity of this pathogen within the state. It is possible that 1 or a few of the 28 STs are already undergoing clonal expansion and are more predominant in certain regions in New Hampshire but that they remain invisible to current surveillance schemes. Hence, future genomic studies should involve a more systematic sampling and active surveillance of patients from health care providers across the state in order to target certain counties and localities if needed (e.g., during outbreaks). Such a statewide strategy across the country will also allow us to precisely define the phylogenetic relationships of C. jejuni cocirculating across the country and map the geographical dispersal of specific clones of interest. Unfortunately, our data set does not include an extensive amount of clinical, phenotypic, or other epidemiological information for each isolate because of how the sampling scheme was set up in the state. This is another important lesson we can learn from this study and apply to future genomic surveillance systems within the state. We strongly advocate for sampling and surveillance schemes of infectious diseases, including *Campylobacter* infections, in the state of New Hampshire that include such pertinent information. Second, only clinical isolates were included in this study, which certainly posed limitations on elucidating the statewide diversity of the pathogen. Asymptomatic individuals may carry a genetically distinct C. jejuni population that remains to be characterized (82, 83). Moreover, because campylobacteriosis is often associated with contaminated food products and exposure to animals (6), whole-genome sequencing of isolates from various sources (agricultural and food production settings, domestic animals, wild animals, and environment) should be a major component of studies of disease ecology and epidemiology. Many reservoirs of C. jejuni are yet to be identified, and bacterial populations from these sources undoubtedly contain many lineages that are yet to be described. Sampling and sequencing from nonclinical sources will provide valuable insights into the sources of horizontally acquired ABR genes, routes and mechanisms of transmission from agricultural and environmental reservoirs to humans, and the genetic bases of bacterial adaptation to specific ecological niches (e.g., host versus nonhost). Widespread application of whole-genome sequencing of foodborne pathogens and other zoonotic diseases across the entire spectrum of the One Health paradigm (84) will, therefore, greatly facilitate public health interventions across multiple sectors. Lastly, next-generation sequencing methods remain imperfect. *In silico* identification of any genetic elements, including resistance genes, relies on high-quality sequencing output. Genome sequencing failures are known to occur with any sequencing platform. Possible sources of errors include a low number of reads, high incidence of unidentified or unreliable nucleotide calls (represented by “N”), high positional bias within the flowcell, and poor overall sequence qualities. The New Hampshire genomes used in our study all have <100 contigs, which is generally satisfactory in many bacterial genome studies. Application of whole-genome sequencing in public health laboratories is expected to improve the quality of sequences given the ongoing and rapid development in sequencing, DNA library preparation, and bioinformatics technologies.

Whole-genome sequencing is a powerful tool that provides timely, accurate, and granular information about a pathogen that can be translated to public health action. The NH DHHS has only recently started sequencing bacterial genomes of select pathogens. This study presents some of the initial results of the state’s initiative to implement whole-genome sequencing in public health laboratories. It is expected that our results will reinforce the need to incorporate pathogen genomics as an integral component of New Hampshire’s disease surveillance, control, clinical decisions, and policy making. Here, we present an analysis of the standing pangenomic variation of clinical C. jejuni within a local region in the United States. We conclude that the diversity of clinical C. jejuni in New Hampshire in 2017 was driven mainly by the coexistence of phylogenetically diverse antibiotic-resistant lineages, widespread geographical mixing, and frequent recombination. Continued genomic surveillance will be necessary to assess how the local population of C. jejuni changes over the long term and in response to changing selective landscapes within the state.

## Supplementary Material

Supplemental file 1

Supplemental file 2

Supplemental file 3

Supplemental file 4

Supplemental file 5

Supplemental file 6

Supplemental file 7

Supplemental file 8

Supplemental file 9
