# Supplementary material for: Genomic Epidemiology and Evolution of Diverse Lineages of Clinical Campylobacter jejuni Cocirculating in New Hampshire, USA, 2017
Source: J Clin Microbiol. 2020 May 26;58(6):e02070-19. doi: 10.1128/JCM.02070-19 (PMC7269400; doi:10.1128/JCM.02070-19)
Supplement: Supplemental file 1 [file JCM.02070-19-s0001.pdf]

SUPPLEMENTAL MATERIAL

Figure S1. Distribution of core and accessory genes in the New Hampshire *C. jejuni* pan-genome

Figure S2. Gene ontology results for the recombined genes identified by fastGEAR

Table S1. Parameters used in each software program to generate data

Table S2. Accession numbers and genome characteristics of *C. jejuni* genomes used in this study

Table S3. Presence-absence matrix for each gene family in the *C. jejuni* pan-genome. 0 = absent, 1 = present

Table S4. Pairwise ANI percentages generated by fastANI

Table S5. MLST profiles for each New Hampshire *C. jejuni* genome

Table S6. Presence-absence matrix for each ABR and virulence gene. 0 = absent, ? = questionable (<95% sequence coverage), 1 = present (≥95% sequence coverage)

Table S7. List of recombined genes and their gene functions identified by fastGEAR

Table S8. Gene ontology results for the recombined genes identified by fastGEAR

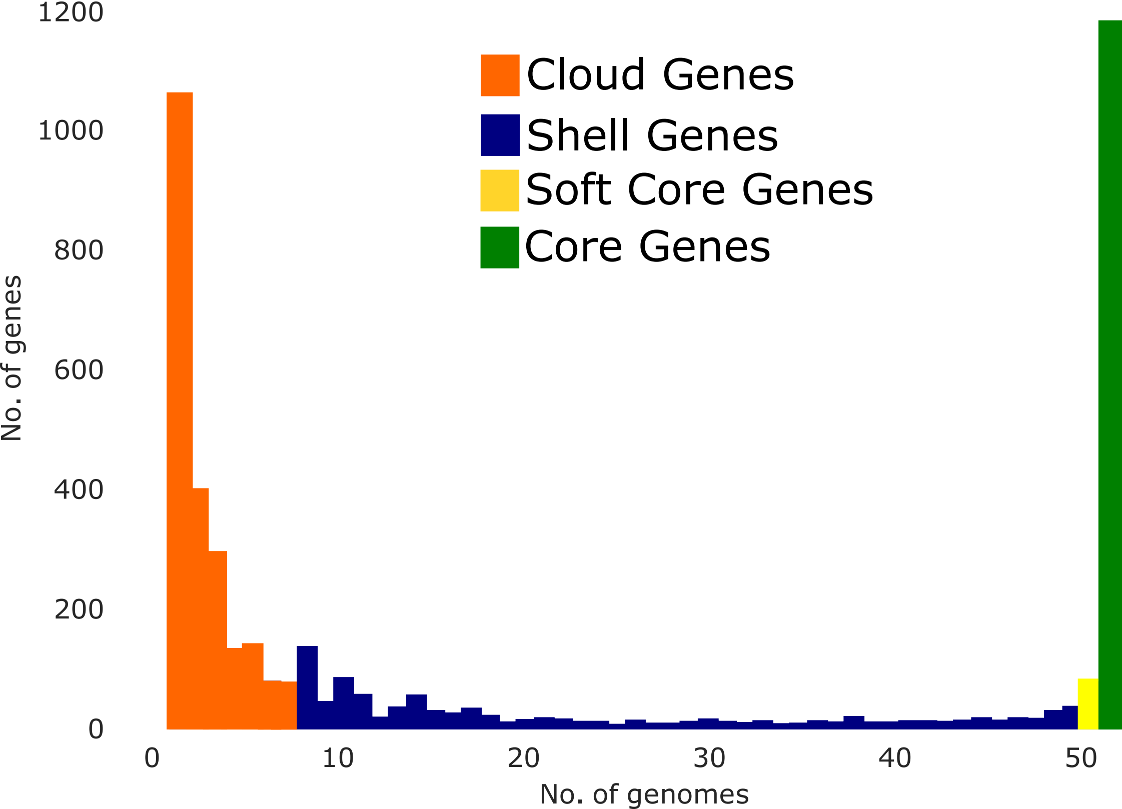

Cloud Genes
Shell Genes
Soft Core Genes
Core Genes
No. of genes
No. of genomes
0
200
400
600
800
1000
1200
0
10
20
30
40
50

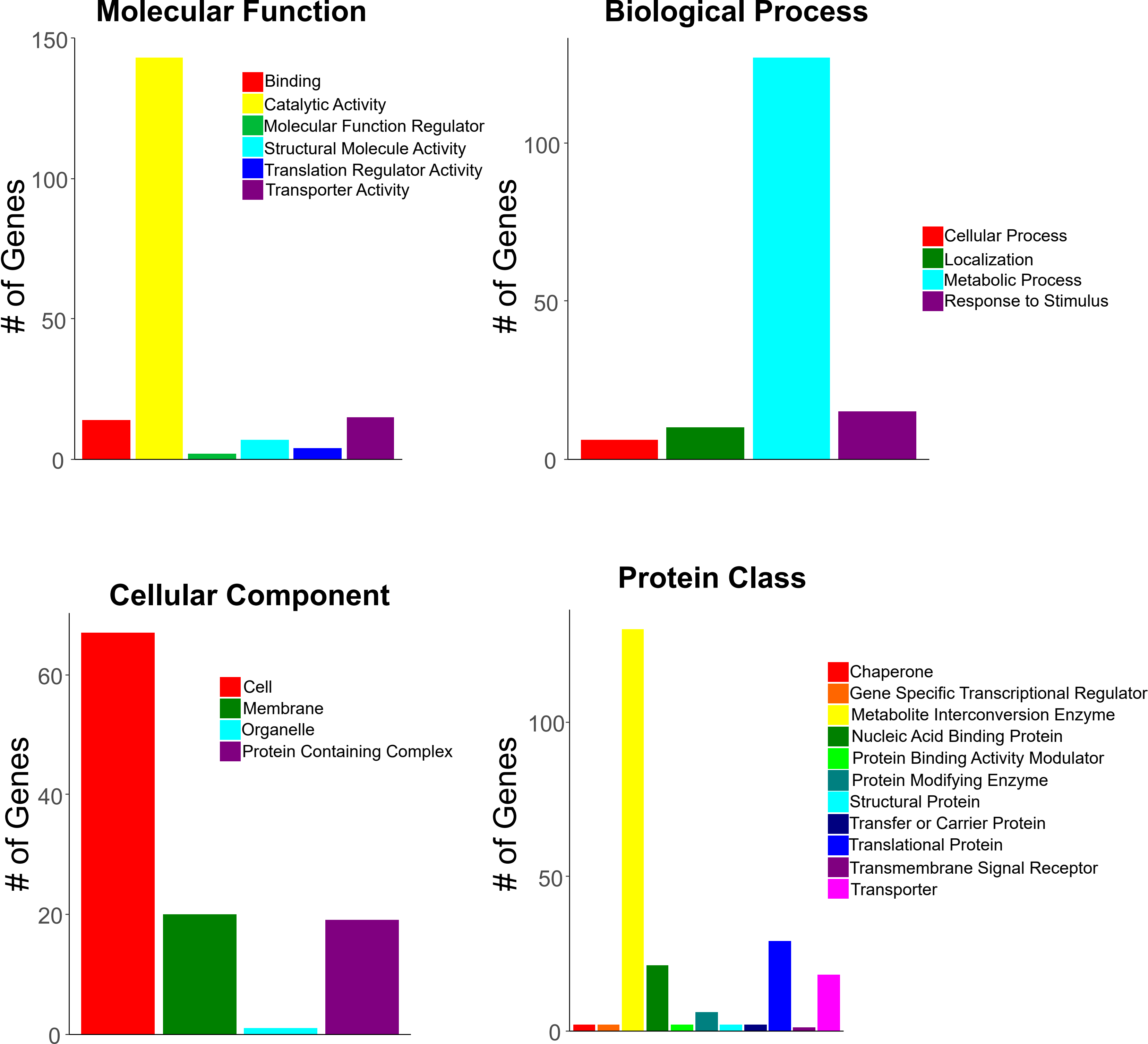
Molecular Function
# of Genes
150
100
50
0
Binding
Catalytic Activity
Molecular Function Regulator
Structural Molecule Activity
Translation Regulator Activity
Transporter Activity
Biological Process
# of Genes
100
50
0
Cellular Process
Localization
Metabolic Process
Response to Stimulus
Cellular Component
# of Genes
60
40
20
0
Cell
Membrane
Organelle
Protein Containing Complex
Protein Class
# of Genes
100
50
0
Chaperone
Gene Specific Transcriptional Regulator
Metabolite Interconversion Enzyme
Nucleic Acid Binding Protein
Protein Binding Activity Modulator
Protein Modifying Enzyme
Structural Protein
Transfer or Carrier Protein
Translational Protein
Transmembrane Signal Receptor
Transporter
